# Putative new biomarkers for renal transporter-mediated drug-drug interactions: Characterization as substrates of organic cation transporter 2, multidrug and toxin extrusion protein 1, and other important drug transporters

**DOI:** 10.1016/j.dmd.2025.100155

**Published:** 2025-08-29

**Authors:** Arne Gessner, Jana Picurová, Lea Englhard, Fabian Müller, Martin F. Fromm, Jörg König

**Affiliations:** 1 Institute of Experimental and Clinical Pharmacology and Toxicology, Friedrich-Alexander-Universität Erlangen-Nürnberg, Erlangen, Germany; 2 FAU NeW – Research Center New Bioactive Compounds, Friedrich-Alexander-Universität Erlangen-Nürnberg, Erlangen, Germany; 3 Boehringer Ingelheim Pharma GmbH & Co. KG, Biberach an der Riss, Germany

**Keywords:** Drug-drug interaction, Transport proteins, Biomarker, Renal drug excretion, OCT2, MATE1

## Abstract

The inhibition of renally expressed transport proteins such as organic cation transporter (OCT) 2 or multidrug and toxin extrusion protein (MATE) 1 can cause clinically relevant drug-drug interactions (DDIs). Endogenous biomarkers have been proposed as tools to characterize the DDI risk of new molecules in drug development. Many previously proposed biomarkers for OCT2/MATE1-mediated DDIs lack specificity and/or sensitivity indicating a need for additional, well characterized biomarkers. Recently, we demonstrated that treatment with cimetidine, a classical OCT/MATE-inhibitor, decreased the renal excretion of serotonin, tyramine, 1-methylhistamine, 5-amino valeric acid betaine, and 4-guanidinobutanoic acid in humans. So far, these compounds are incompletely characterized as substrates of OCT2, MATE1, and other drug transporters. We therefore used established cell models overexpressing OCT2 and/or MATE1, and cell models for other clinically important drug transporters (organic anion transporters 1 and 3, organic anion transporting polypeptides 1B1 and 1B3, and P-glycoprotein) to investigate the cellular uptake and/or vectorial transport of these 5 putative biomarkers. The in vitro results show that serotonin, tyramine, 1-methylhistamine, 5-amino valeric acid betaine, and 4-guanidinobutanoic acid are substrates of OCT2 and/or MATE1, supporting that the in vivo effect of cimetidine is due to inhibition of these transporters. Based on their transport by OCT2 and/or MATE1 compared to the minimal transport by other drug transporters and the in vivo effects of classical transport protein inhibitors in healthy volunteers, serotonin and 1-methylhistamine appear to be the most promising candidates for further validation as endogenous biomarkers for the early detection of clinically relevant OCT2- and MATE1-mediated renal DDIs.

**Significance Statement:**

This study characterizes 5 endogenous metabolites as substrates of the renally expressed transport proteins organic cation transporter (OCT) 2, multidrug and toxin extrusion protein (MATE) 1, and other important drug transporters. These transport proteins are known as important contributors to clinically observed drug-drug interactions. Of the respective 5 compounds, serotonin and 1-methylhistamine are the most promising candidates to be further investigated as biomarkers for interactions via OCT2/MATE1, which could improve drug-drug interaction assessment during clinical drug development.

## Graphical abstract

**Figure:**
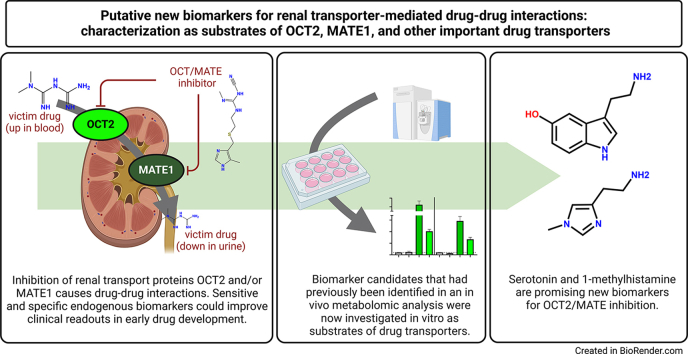

## 1 Introduction

Transport proteins are fundamental determinants of pharmacokinetics.^1^, ^2^, ^3^ Many desired or adverse drug effects depend on transport processes across cellular membranes.^1^, ^2^, ^3^, ^4^, ^5^ Transport proteins expressed in hepatocytes and proximal tubule cells of the kidney are of considerable importance for pharmacokinetics and the excretion of drugs or drug metabolites. If 2 or more drugs interact with the same transport protein, there is a potential for clinically relevant drug-drug interactions (DDIs), as a perpetrator drug may alter pharmacokinetics of a victim drug.^1^^,^^4^^,^^5^ Therefore, regulatory authorities require a thorough assessment for potential transporter-mediated DDIs during drug development, as described in the ICH M12 guideline on drug interaction studies.^6^^,^^7^ These recommendations focus on several transport proteins such as, organic cation transporter (OCT) 2 (gene symbol *SLC22A2*), multidrug and toxin extrusion protein (MATE) 1 (gene symbol *SLC47A1*), organic anion transporters (OAT) 1 and OAT3 (gene symbols *SLC22A6* and *SLC22A8*, respectively), organic anion transporting polypeptides (OATP) 1B1 and OATP1B3 (gene symbols *SLCO1B1* and *SLCO1B3*, respectively), and P-glycoprotein (P-gp, gene symbol *ABCB1*).

During drug development, new drug candidates are tested in vitro as substrates and inhibitors of clinically relevant transport proteins.^5^, ^6^, ^7^ Based on these results, subsequent clinical studies on DDIs in healthy volunteers may be required, which in many cases prove the absence of clinically relevant interactions of the investigated drug combination.^8^, ^9^, ^10^, ^11^ These time and cost consuming procedures put study participants at risk due to the treatment with a new molecular entity in clinical development. In order to reduce risks for study participants and improve early clinical readouts on the potential for DDIs, endogenous compounds are currently investigated as biomarkers for putative transporter-mediated DDIs.^8^^,^^9^^,^^12^, ^13^, ^14^ Especially, biomarkers for renally expressed drug transporters are intensively discussed.^8^^,^^9^^,^^12^^,^^13^^,^^15^^,^^16^ However, in a study with healthy volunteers investigating previously proposed biomarkers for DDIs mediated by the renally expressed drug transporters OCT2 and MATE1, we found that many of them lack sensitivity and/or specificity, which are important properties of valid transporter biomarkers.^15^

In a metabolomic analysis using human plasma and urine samples, we recently identified new candidates for biomarkers of DDIs via renally expressed transport proteins.^16^ The endogenous metabolites serotonin, tyramine, 1-methylhistamine, 5-amino valeric acid betaine, and 4-guanidinobutanoic acid are of particular importance, as their renal excretion was considerably reduced upon treatment with the known OCT/MATE inhibitor cimetidine, in comparison to treatment with other classical transport protein inhibitors, ie, probenecid (inhibitor of OATs), verapamil (inhibitor of P-gp), and rifampin (inhibitor of OATPs) (Table 1).^16^ To the best of our knowledge, of those 5 new biomarker candidates, only serotonin, tyramine, and 4-guanidinobutanoic acid have been partially described as substrates of OCT2 and/or MATE1,^17^^,^^18^ and data regarding the transport by other clinically relevant drug transporters are rare.

**TABLE 1:** Comparison of in vivo and in vitro results for putative biomarkers

In vivo – in vitro consistency^d^	In Vivo^a^ Change of Renal Excretion	In Vitro^b^		In Vivo^a^ Change of Renal Excretion	In Vitro^b^	In Vivo^a^ Change of Renal Excretion	In Vitro^b^		In Vivo^a^ Change of Renal Excretion	In Vitro^b^		Potential as Biomarker for Transporter-Mediated DDI
	Cimetidine	OCT2	MATE1	Verapamil	P-gp	Rifampin	OATP1B1	OATP1B3	Probenecid	OAT1	OAT3	
**Serotonin**	–2.89^c^	++	++	0.00	-	–0.06	-	-	0.06	-	0	**High** (specific and sensitive in vitro and in vivo)

In vivo – in vitro consistency	✓			✓		✓			÷		

**Tyramine**	–3.00^c^	++	++	–0.22	-	–0.11	0	-	–0.82	-	0	**Limited** (uptake in HEK-OAT3 and in vivo effect of probenecid)

In vivo – in vitro consistency	✓			✓		÷			✓		

**1-Methylhistamine**	–2.01^c^	++	++	0.03	0	–0.07	-	-	–0.25	-	-	**High** (specific and sensitive in vitro and in vivo)

In vivo – in vitro consistency	✓			÷		✓			✓		

**5-Amino valeric acid betaine**	–4.28^c^	+	+	–1.00	-	–0.21	0	0	–0.56^c^	-	-	**Unclear** (endogenous background in vitro)

In vivo – in vitro consistency	✓			✓		÷			×		

**4-Guanidinobutanoic acid**	–2.06^c^	+	+	–0.28	0	–0.15	-	-	–0.18	0	-	**Unclear** (endogenous background in vitro)

In vivo – in vitro consistency	✓			÷		✓			÷		

a In vivo: numbers indicate median log2-fold change after treatment with the respective inhibitor. In vivo data are from a previously published metabolomic study in humans, investigating the effect of the transport protein inhibitors cimetidine, verapamil, rifampin, and probenecid.

b In vitro: ++: substrate; +: most likely substrate (complication due to intracellular background); 0: significant uptake in HEK cells, but less than 300% of respective control cells or significant reduction of transcellular transport in Caco-2 cells, but reduction not below 50% of respective control without valspodar; -: no significant uptake and/or transcellular transport.

c Sensitive changes are indicated (for original data and criteria for sensitivity see Gessner et al^16^).

d In vivo – in vitro consistency: ✓: consistent (putative biomarkers sensitively changed due to inhibitor treatment in vivo are substrates of the respective transport proteins in vitro, and compounds that are not substrates for transport proteins in vitro are not sensitive to the respective inhibitor treatment); ÷: partly consistent (in vitro significant uptake/transport by transport protein, but no sensitive change in vivo); ×: not consistent (sensitive change in vivo, but not substrate of respective transport proteins).

To gain further insights into the role of OCT2 and/or MATE1 in renal excretion of the 5 putative biomarkers and to further support the hypothesis that our previous finding of reduced renal excretion in vivo due to cimetidine treatment is caused by inhibition of transport proteins and not by other effects of cimetidine, we performed in vitro experiments using established human embryonic kidney 293 (HEK) cell lines stably overexpressing these transport proteins and mass spectrometric quantification to (further) characterize serotonin, tyramine, 1-methylhistamine, 5-amino valeric acid betaine, and 4-guanidinobutanoic acid as substrates of OCT2 and MATE1. In addition, single- and double-transfected Madin-Darby canine kidney II (MDCK) cells overexpressing OCT2 and/or MATE1 were used to investigate vectorial, basal to apical transport (simulating transporter-mediated secretion in the proximal renal tubule). To verify the specificity of OCT2- and MATE1-mediated transport, uptake experiments into HEK cells overexpressing other drug transporters were performed (OAT1, OAT3, OATP1B1, or OATP1B3), as well as vectorial transport assays in Caco-2 cells investigating P-gp–mediated transport. The results of the present work elucidate the molecular background of the observed effect of cimetidine on renal excretion of these endogenous compounds. Moreover, the results serve as an in vitro assessment and qualification of these substances as new biomarkers for OCT2/MATE1-mediated renal DDIs.

## 2 Materials and methods

### 2.1 Reagents and chemicals

Acetonitrile, methanol (MeOH), water, and formic acid (all LC/MS-grade) were purchased from VWR chemicals. Ammonium formate (LC/MS-grade), [^2^H_5_]-tryptophan, tyramine-HCl, serotonin-HCl, 4-guanidinobutanoic acid, 1-methylhistamine-2HCl, cimetidine, digoxin, and valspodar were purchased from Sigma-Aldrich. 1-Methyl-4-phenylpyridinium iodide (MPP^+^), *p*-aminohippuric acid (PAH), and estrone 3-sulfate (E3S) were from Biomol. 5-amino valeric acid betaine and [^2^H_3_]-1-methylhistamine-2HCl was from MedChemtronica. [^2^H_3_]-N8-acetylspermidine-2HCl was from Santa Cruz Biotechnology. Bromosulfophthalein (BSP) was from AppliChem. [^3^H]-BSP (10.2 Ci/mmol) and [^3^H]-serotonin (80 Ci/mmol) were from Hartmann Analytic. [^3^H]-MPP^+^ iodide (80 Ci/mmol), [^3^H]-PAH (40 Ci/mmol), [^3^H]-E3S (50 Ci/mmol), and [^3^H]-digoxin (20 Ci/mmol) were from American Radiolabeled Chemicals, Inc.

Poly-D-lysine hydrobromide and sodium butyrate were purchased from Sigma-Aldrich. Cellstar 12-well cell culture plates and ThinCert Inserts (12 well, pore size 0.4 *μ*m, translucent, filter area 1.1 cm^2^) were from Greiner Bio-One GmbH or Sarstedt AG & Co. KG. Sodium butyrate was from Merck KGaA. Cell culture media supplements were obtained from Thermo Fisher Life Technologies GmbH. All other chemicals and reagents, unless stated otherwise, were obtained from Carl Roth GmbH + Co. KG.

### 2.2 Cell culture

Generation, characterization, and cell culture conditions of the used cell lines have been described previously.^19^, ^20^, ^21^, ^22^, ^23^ HEK-OAT1 and HEK-OAT3 cells were newly established as described below. Uptake assays were performed using HEK or MDCK cells transfected with an empty vector for control cells (vector control [VC]), or stably overexpressing human OCT2 and/or MATE1. Furthermore, HEK cells stably overexpressing human OAT1, OAT3, OATP1B1, or OATP1B3 and parental Caco-2 cells were used for the assessment of P-gp–mediated transport. HEK and MDCK cells were cultured in minimal essential medium containing 10% heat-inactivated fetal bovine serum, 100 U/L penicillin, 100 *μ*g/mL streptomycin, and G418 (800 *μ*g/mL) and/or hygromycin B (260 *μ*g/mL). Caco-2 cells were cultivated in Dulbecco’s modified Eagle’s medium (low glucose, supplemented with L-alanyl-L-glutamine and pyruvate) with 10% heat-inactivated fetal bovine serum, 100 U/L penicillin, 100 *μ*g/mL streptomycin, and 0.1 mM nonessential amino acids. All cells were cultured at 37 °C and 5% CO_2_ with subculture as required. Trypsin (0.05%)-EDTA (0.02%) solution was used to detach cells. All cell culture media and supplements were obtained from Thermo Fisher Life Technologies GmbH.

**Fig. 1:**
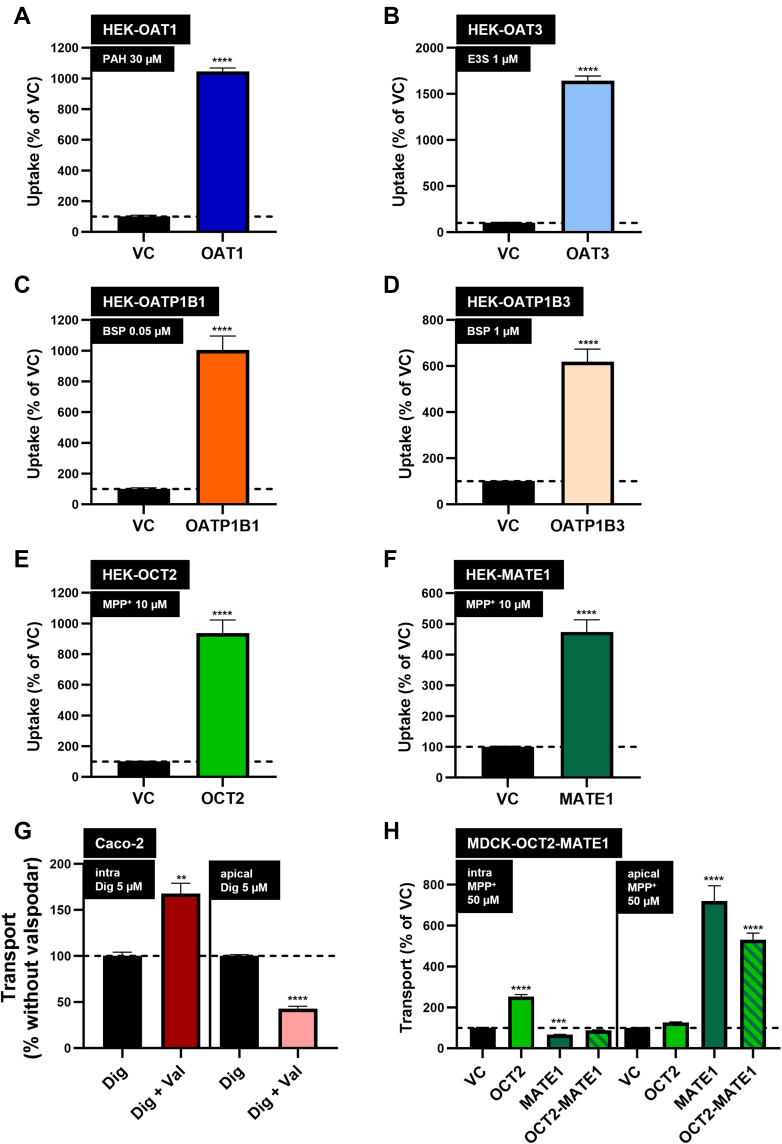
Control uptake experiments with known substrates. Uptake and transcellular transport of known substrates (A) PAH for HEK-OAT1, (B) E3S for HEK-OAT3, (C, D) BSP for HEK-OATP1B1 and HEK-OATP1B3, (E, F, H) MPP^+^ for HEK-OCT2, HEK-MATE1, and MDCK cells and (G) digoxin (Dig) for P-gp in Caco-2 cells. Substrate concentrations are given in the respective subtitles in the figure. Uptake or transcellular transport was measured after 5 minutes of incubation for HEK cells, after 60 minutes for MDCK cells, and after 240 minutes for Caco-2 cells in the presence and absence of the P-gp inhibitor valspodar (2 *μ*M). Data are shown as mean ± SEM resulting from 4 biological replicates, ∗∗*P* < .01 vs VC/no valspodar; ∗∗∗*P* < .001 vs VC ∗∗∗∗*P* < .0001 vs VC/no valspodar (two-tailed unpaired Student’s *t* test). Val, added valspodar; intra, intracellular accumulation; apical, transcellular transport from the basal to the apical compartment.

HEK cells were used to investigate cellular uptake, whereas polarized monolayers of MDCK and Caco-2 cells were used to study intracellular accumulation and transcellular transport from the basal to the apical compartment in a transwell setup. For all experiments, MPP^+^ for OCT2 and MATE1, PAH for OAT1, E3S for OAT3, BSP for OATP1B1 and OATP1B3, and digoxin for P-gp served as positive control substrates^6^^,^^7^ as shown in Fig. 1. All of these substances were used in a [^3^H]-radiolabeled form as previously described.^19^

### 2.3 Establishment of stably-transfected HEK cells recombinantly overexpressing human OAT1 or human OAT3

The *SLC22A6* (encoding human OAT1) and *SLC22A8* (encoding human OAT3) cDNAs were cloned by a polymerase chain reaction–based approach. Commercially available kidney total RNA was used as a template for the amplification of both cDNAs. For the *SLC22A6* cDNA, the primer pair oOAT1-5′.for (5′-ggc cca atg gcc ttt aat gac-3′) and oOAT1-RT.rev (5′-agt cct cag agt cca ttc tt-3′) was used; for the *SLC22A8* cDNA, the primer pair oOAT3-5′.for (5′-cca gtg cca tga cct tct cg-3′) and oOAT3-RT.rev (5′-gtc ctc agc tgg agc cca g-3′) was used. Both amplified cDNA fragments were cloned into the vector pCR2.1.TOPO (Thermo Fisher Life Technologies GmbH) and sequenced. Point mutations were corrected using the QuikChange Multi Site-directed Mutagenesis Kit (Agilent) according to the manufacturer's instructions resulting in cDNAs encoding proteins entirely identical to the proteins in the reference sequence (OAT1: NM_004790.4; OAT3: NM_004254.3). Finally, the *SLC22A6* cDNA was subcloned into the expression vector pcDNA3.1/Hy(–), resulting in the plasmid pOAT1.31 whereas the *SLC22A8* cDNA was subcloned into the expression vector pcDNA3.1(+) resulting in the plasmid pOAT3.31 (both vectors from Life Technologies GmbH). Using both plasmids, stably transfected HEK cells overexpressing human OAT1 or OAT3 were established as described.^24^ HEK cell clones with the highest expression of the *SLC22A6* or the *SLC22A8* mRNA were selected by quantitative reverse-transcription polymerase chain reaction and functionally characterized by standardized uptake assays using PAH (30 *μ*M) or E3S (1 *μ*M) as prototypic substrates for OAT1 (HEK-OAT1) or OAT3 (HEK-OAT3), respectively, as described below.

### 2.4 Uptake transport assays with HEK cells

Uptake assays into HEK cells stably overexpressing transport proteins and their respective VC were performed as previously described with minor modifications.^19^ In brief, cells were seeded in poly-D-lysine–coated (0.1 mg/mL) 12-well plates with a density of 7 × 10^5^ cells per well. Twenty-four hours after seeding, cells were induced with 10 mM sodium butyrate to enhance protein expression. Transport experiments were performed 48 hours after seeding. Cells were washed once with transport buffer at 37 °C. Afterward, cells were incubated with transport buffer containing (putative) substrates at the desired concentration at 37 °C for 5 minutes. Inhibition of OCT2/MATE1-mediated transport was investigated by adding 50 *μ*M of cimetidine to the above-mentioned transport buffer containing putative substrates. A mixture of unlabeled and [^3^H]-labeled substrate was used for control experiments. The buffer for the transport experiments contained 142 mM NaCl, 5 mM KCl, 1 mM K_2_HPO_4_, 1.2 mM MgSO_4_, 1.5 mM CaCl_2_, 5 mM glucose, and 12.5 mM HEPES. The pH value was set to 7.3, except for HEK-MATE1 cells and their respective VC, in which pH was set to 8.0.

At the end of the incubation period, cells were put on ice, the transport buffer was removed, and cells were rinsed thrice with ice-cold 0.9% NaCl in water. Then, cells were lysed with 700 *μ*L of an ice-cold mixture of MeOH and water (80:20) containing the recovery standards [^2^H_5_]-tryptophan (1 *μ*g/mL) and [^2^H_3_]-N8-acetylspermidine (0.1 *μ*g/mL). Subsequently, lysates were analyzed with liquid chromatography coupled to tandem mass spectrometry (LC-MS).

In the control experiments, cells were rinsed thrice with ice-cold transport buffer (pH 7.3 or pH 8.0) and lysed with SDS (0.2%). The amount of radioactivity in the cell lysates was measured using liquid scintillation counting (Tricarb 2800; Perkin Elmer Life and Analytical Sciences Inc), and protein concentrations were measured by bicinchoninic acid assay (Pierce BCA protein assay kit; Thermo Fisher Life Technologies GmbH).

To determine the concentration-dependent transport of serotonin and 1-methylhistamine by OCT2 and MATE1, cells were incubated for 5 minutes with substrate concentrations of 10, 50, 100, 250, 500, and 1000 *μ*M. Additionally, concentration-dependent transport of 1-methylhistamine by OCT2 was analyzed for 5 minutes using 200, 300, 400, 1250, 1500, and 2000 *μ*M of substrate. Divergent from the description of the initial cellular uptake experiments above, serotonin in transport buffer was adjusted to 1 *μ*Ci/*μ*L using [^3^H]-radiolabeled serotonin and measured using liquid scintillation counting, and for 1-methylhistamine, cell lysis was performed with 700 *μ*L of an ice-cold mixture of MeOH and water (80:20) containing the recovery standard [^2^H_3_]-1-methylhistamine (0.025 *μ*g/mL) and subsequently analyzed by LC-MS.

### 2.5 Cellular uptake and transcellular transport assays with MDCK and Caco-2 cells

The intracellular accumulation and transcellular, basal to apical transport experiments were performed as previously described in monolayers of polarized single-transfected (MDCK-OCT2, MDCK-MATE1) and double-transfected (MDCK-OCT2-MATE1) MDCK cells, MDCK control cells (MDCK-VC), or Caco-2 cells.^22^ In brief, cell monolayers were grown in cell culture inserts on porous membranes. For MDCK-VC, MDCK-OCT2 and Caco-2, 5 × 10^5^ cells were used per well; for MDCK-OCT2-MATE1, 6 × 10^5^ cells were used, and for MDCK-MATE1, 8 × 10^5^ cells were used per well. Cells were grown to confluence for 72 hours, induced with 10 mM sodium butyrate 48 hours after seeding for 24 hours (MDCK only), and subsequently used for transport experiments.

In general, after the cell culture medium was removed from both sides of the monolayers, cells were washed at 37 °C with transport buffer of the same composition as described in the previous section. Experiments were started by replacing the medium in the basal compartment with medium containing the putative biomarkers or [^3^H]-labeled positive control substrates. The pH of the buffer on the basal side was 7.3 and 6.5 on the apical side for MDCK cells, whereas it was 7.3 on either side for Caco-2 cells. Cells were incubated at 37 °C and the buffer from the apical compartment was taken after 60 minutes for MDCK cells and after 240 minutes for Caco-2 cells. In case of Caco-2 cells, endogenous P-gp was inhibited by adding 2 *μ*M of valspodar to the basal compartment. To measure the cellular accumulation of the test compounds, the remaining medium was removed at the end of the incubation period and the cell monolayers were rapidly rinsed 3 times with ice-cold 0.9% NaCl in water. Filters were detached from the chambers and placed into 500 *μ*L of 80% MeOH containing the aforementioned recovery standards to lyse cells for subsequent LC-MS analysis or into 600 *μ*L of 0.2% SDS (control experiments only). In the control experiments, the radioactivity of the collected apical medium and the solubilized cell monolayers and protein concentrations of the cell lysates were measured in the same way as described for the experiments with HEK cells.

### 2.6 Determination of intracellular background in HEK, MDCK, and Caco-2 cells

The intracellular background of all 5 compounds in HEK cells at the beginning of the uptake experiments was investigated 48 hours after seeding without incubation with transport buffer (ie, 0 minutes). For this purpose, cell culture medium was removed, and the cells were rinsed thrice with ice-cold 0.9% NaCl in water. Then, cells were lysed with ice-cold 80% MeOH containing the aforementioned recovery standards. In addition, the impact of a 5-minute incubation period with transport buffer without added substance was investigated. In monolayers of MDCK and Caco-2 cells, endogenous intracellular concentrations of compounds and their transport to the apical compartment was investigated in cells that were not incubated with the respective putative biomarker (eg, serotonin was tested in cells incubated with tyramine, 1-methylhistamine, 5-amino valeric acid betaine, and 4-guanidinobutanoic acid) after the 60 minutes (MDCK) or 240 minutes (Caco-2) incubation period.

### 2.7 LC-MS analysis

Analysis was performed on an LC system (Agilent 1100; Agilent Technologies) coupled to a triple quadrupole-MS (API 4000; Applied Biosystems) featured with an electrospray ion source operated in positive ionization mode. Chromatography was carried out isocratically at a flow rate of 0.3 mL/min. The eluent consisted of 625 mg/L ammonium formate in acetonitrile-water (80:20), which was adjusted to pH 3.5 with formic acid. The column was a 2.1 × 100 mm Acquity UPLC BEH Amide column (1.7 *μ*m) equipped with a 2.1 × 5 mm guard column (both from Waters). The column temperature was set to 40 °C and the run time to 6 minutes. Analyte-specific multiple reaction monitoring transitions and retention times are shown in Table 2. For sample preparation, methanolic cell lysates were centrifuged at 24000 *g* and 4 °C for 5 minutes using an Eppendorf 5427R centrifuge (Eppendorf), and 200 *μ*L of the supernatant were pipetted into a glass vial. After evaporation to dryness under a gentle stream of nitrogen at 30 °C, the residue was reconstituted in 300 *μ*L of eluent and 10 *μ*L were injected for LC-MS analysis. For samples from the apical compartment of experiments with MDCK and Caco-2 cells, 60 *μ*L were mixed with 240 *μ*L of MeOH containing recovery standards. After centrifugation, 200 *μ*L of supernatant were prepared as described.

**Table 2:** Retention times and multiple reaction monitoring transitions for LC-MS analysis All peaks consistently had a signal-to-noise ratio >10.

Analyte	RT *min*	Q1	Q3
5-Amino valeric acid betaine (quantifier)	1.3	160.1	55.1
5-Amino valeric acid betaine (qualifier)			101.1
Tyramine (quantifier)	1.4	138.1	121.1
Tyramine (qualifier)			103.1
Serotonin (quantifier)	1.5	177.1	115.1
Serotonin (qualifier)			132.1
4-Guanidinobutanoic acid (quantifier)	1.6	146.1	87.1
4-Guanidinobutanoic acid (qualifier)			86.1
[^2^H_5_]-Tryptophan	1.7	210.1	192.2
1-Methylhistamine (quantifier)	3.8	126.1	68.1
1-Methylhistamine (qualifier)			97.0
[^2^H_3_]-1-Methylhistamine	3.8	128.9	68.0
[^2^H_3_]-N8-Acetylspermidine	4.7	191.2	117.1

RT, retention time; Q1, precursor ion; Q3, product ion.

### 2.8 Data analysis

After LC-MS analysis, area ratios were calculated between the signal area found for the test compound and the respective recovery standard, which was [^2^H_5_]-tryptophan for serotonin, tyramine, 5-amino valeric acid betaine, and 4-guanidinobutanoic acid, and [^2^H_3_]-N8-acetylspermidine for 1-methylhistamine in initial experiments. All peaks consistently had a signal-to-noise ratio >10. Uptake or transcellular transport were calculated by dividing the area ratio (test compound/recovery standard) found in individual samples by the mean area ratio found in VC. To determine the concentration-dependent transport, absolute quantification methods meaning liquid scintillation counting in the case of serotonin and LC-MS calibration curves for 1-methylhistamine (area ratio of 1-methylhistamine/[^2^H_3_]-1-methylhistamine) were employed. Data are presented in percentage of respective controls (which were set to 100%) or in pmol × mg protein^–1^ × min^–1^ for concentration-dependent transport as mean ± SEM. All results were normalized to the mean protein concentrations of the cell lysates in the respective control experiments.

Experiments with putative biomarkers in HEK-OCT2 or HEK-MATE1 cells were performed with 6 independent biological replicates, whereas all other experiments involving HEK, MDCK, or Caco-2 cells were performed with 4 independent biological replicates. Pairwise comparisons were analyzed for statistical significance with two-tailed unpaired Student’s *t* test and multiple comparisons by one-way ANOVA with subsequent Tukey-Kramer multiple comparison test by using GraphPad Prism 10.0.2 (GraphPad Software). A value of *P* < .05 was considered as statistically significant. K_m_ and V_max_ values were determined with Michaelis-Menten enzyme kinetics curve fit using GraphPad Prism 10.0.2.

## 3 Results

A comparison of previously published in vivo metabolomic data with the present in vitro results for putative biomarkers of OCT2/MATE1-mediated renal DDIs is shown in Table 1. Results of control experiments for all investigated transporters with well characterized radiolabeled substrates are shown in Fig. 1.

### 3.1 OCT2-mediated uptake

**Fig. 2:**
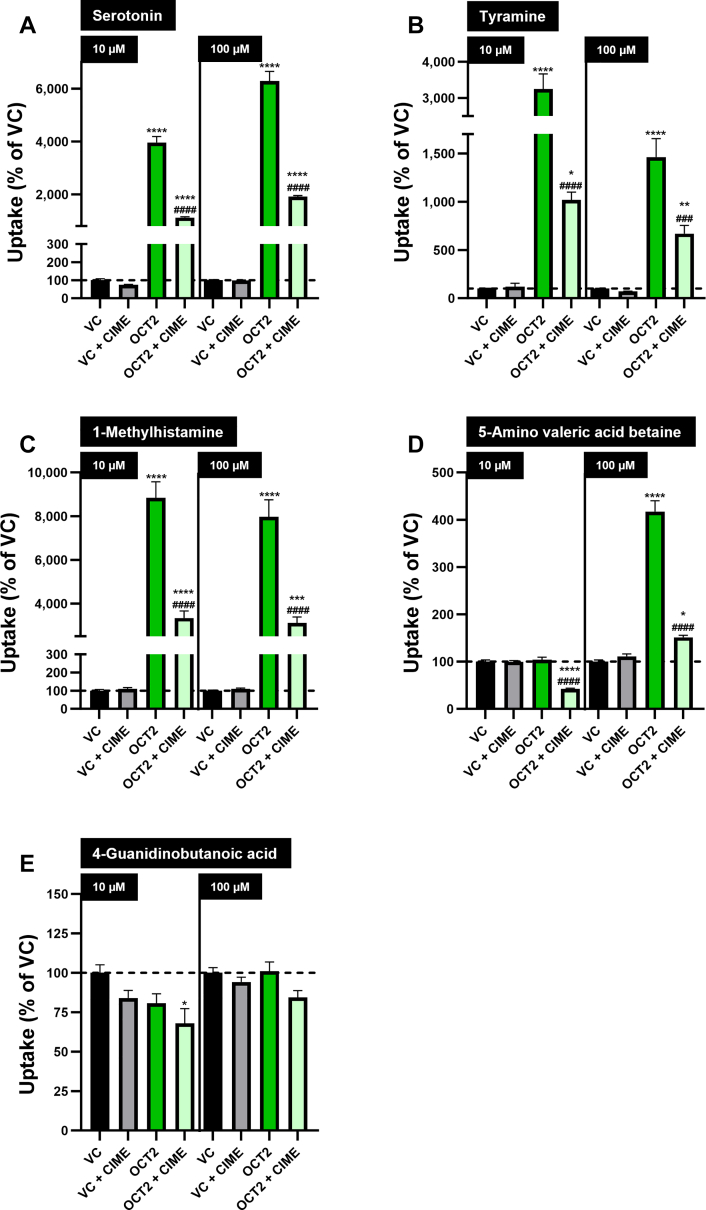
Uptake of 5 putative biomarkers for OCT2/MATE1-mediated DDIs into HEK-OCT2 and HEK-VC cells. Uptake of (A) serotonin, (B) tyramine, (C) 1-methylhistamine, (D) 5-amino valeric acid betaine, and (E) 4-guanidinobutanoic acid (10 and 100 *μ*M) was measured after 5 minutes of incubation in the presence and absence of the inhibitor cimetidine (CIME) (50 *μ*M). Uptake into HEK-VC cells is set to 100% and is indicated by a dashed horizontal line. Data are shown as mean ± SEM resulting from 6 biological replicates. ∗*P* < .05 vs HEK-VC; ∗∗*P* < .01 vs HEK-VC; ∗∗∗*P* < .001 vs HEK-VC; ∗∗∗∗*P* < .0001 vs HEK-VC; ###*P* < .001 vs HEK-OCT2; ####*P* < .0001 vs HEK-OCT2; “no symbol” *P* > .05 (one-way ANOVA with subsequent Tukey-Kramer multiple comparison test; only comparisons between HEK-VC vs HEK-OCT2, HEK-VC vs HEK-OCT2 + CIME, and HEK-OCT2 vs HEK-OCT2 + CIME are shown).

Uptake of serotonin (Fig. 2A), tyramine (Fig. 2B), and 1-methylhistamine (Fig. 2C) was significantly higher in HEK-OCT2 cells compared to HEK-VC cells (eg, 10 *μ*M: 3960%, 3245%, and 8844% for serotonin, tyramine, and 1-methylhistamine, respectively). For these 3 putative biomarkers, uptake in HEK-OCT2 cells was significantly reduced by adding the OCT2 inhibitor cimetidine. In experiments with 100 *μ*M substrate concentration, 5-amino valeric acid betaine was found in significantly higher intracellular concentrations in HEK-OCT2 cells compared to HEK-VC cells and was significantly reduced due to addition of cimetidine. However, at 10 *μ*M, 5-amino valeric acid betaine uptake was significantly reduced by cimetidine in HEK-OCT2 cells, but not in HEK-VC cells (Fig. 2D). No significant difference was detected between HEK-OCT2 and HEK-VC cells for 4-guanidinobutanoic acid (Fig. 2E).

### 3.2 MATE1-mediated uptake

**Fig. 3:**
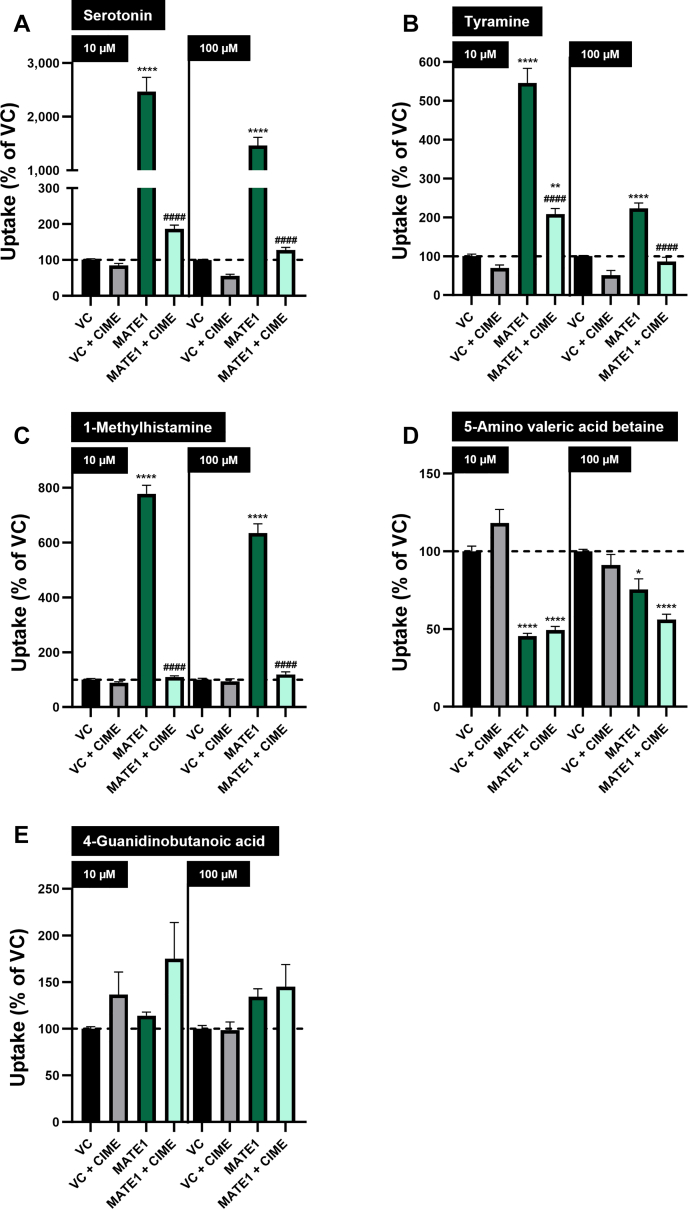
Uptake of 5 putative biomarkers for OCT2/MATE1-mediated DDIs into HEK-MATE1 and HEK-VC cells. Uptake of (A) serotonin, (B) tyramine, (C) 1-methylhistamine, (D) 5-amino valeric acid betaine, and (E) 4-guanidinobutanoic acid (10 and 100 *μ*M) was measured after 5 minutes of incubation in the presence and absence of the inhibitor cimetidine (CIME) (50 *μ*M). Uptake into HEK-VC cells is set to 100% and is indicated by a dashed horizontal line. Data are shown as mean ± SEM resulting from 6 biological replicates, ∗*P* < .05 vs HEK-VC; ∗∗*P* < .01 vs HEK-VC; ∗∗∗∗*P* < .0001 vs HEK-VC; ####*P* < .0001 vs HEK-MATE1; “no symbol” *P* > .05 (one-way ANOVA with subsequent Tukey-Kramer multiple comparison test; only comparisons between HEK-VC vs HEK-MATE1, HEK-VC vs HEK-MATE1 + CIME, and HEK-MATE1 vs HEK-MATE1 + CIME are shown).

Similar to OCT2, serotonin, tyramine, and 1-methylhistamine were taken up into HEK-MATE1 cells at a significantly higher rate compared to HEK-VC cells (eg, 10 *μ*M: 2469%, 545% and 778% for serotonin, tyramine and 1-methylhistamine, respectively) (Fig. 3, A, B, and C). This uptake was significantly reduced by adding cimetidine. Interestingly, in HEK-MATE1 cells, intracellularly detected 5-amino valeric acid betaine was significantly lower than in HEK-VC cells for both concentrations tested (Fig. 3D). As experienced for OCT2-mediated uptake, no MATE1-mediated uptake could be detected for 4-guanidinobutanoic acid (Fig. 3E).

### 3.3 Intracellular accumulation and transcellular transport in monolayers of single- and double-transfected MDCK cells

**Fig. 4:**
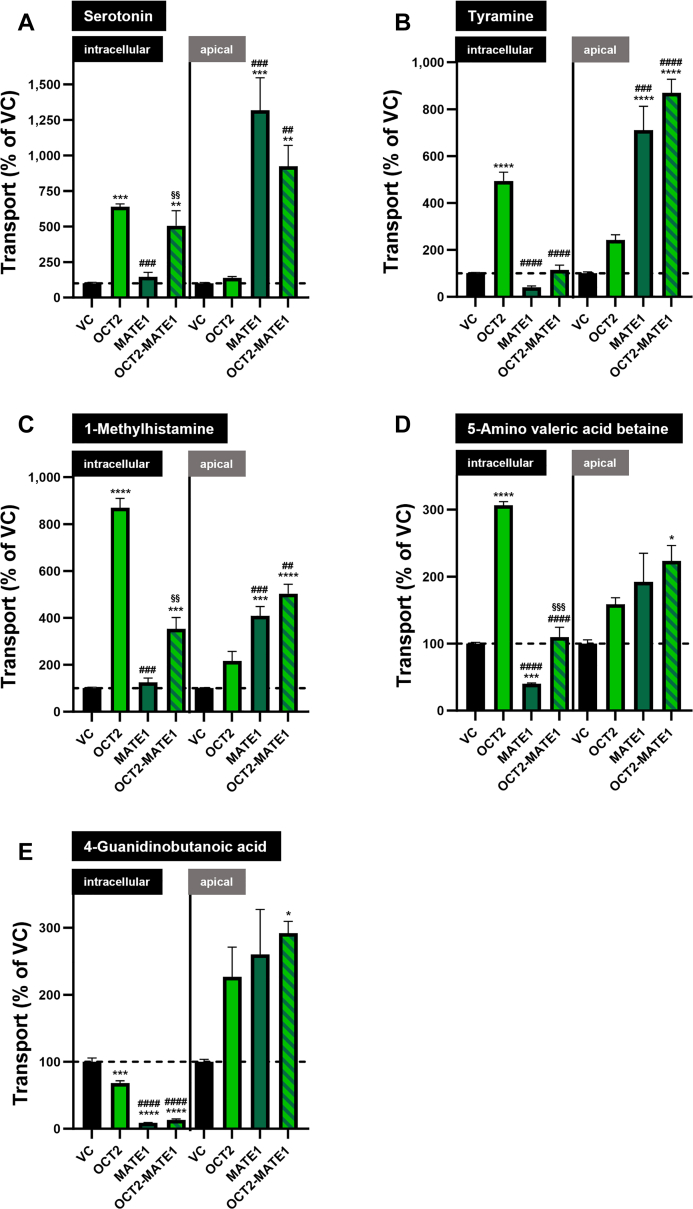
Uptake and transcellular transport of 5 putative biomarkers for OCT2/MATE1-mediated DDIs in MDCK-VC, MDCK-OCT2, MDCK-MATE1, and MDCK-OCT2-MATE1 cells. Intracellular accumulation (intracellular) and vectorial transport from the basal to the apical compartment (apical) of (A) serotonin, (B) tyramine, (C) 1-methylhistamine, (D) 5-amino valeric acid betaine, and (E) 4-guanidinobutanoic acid (10 *μ*M) was measured after 60 minutes. Accumulation/transport in MDCK-VC cells is set to 100% and is indicated by a dashed horizontal line. Data are shown as mean ± SEM resulting from 4 biological replicates, ∗*P* < .05 vs MDCK-VC; ∗∗ *P* < .01 vs MDCK-VC; ∗∗∗*P* < .001 vs MDCK-VC; ∗∗∗∗*P* < .0001 vs MDCK-VC; ##*P* < .01 vs MDCK-OCT2; ###*P* < .001 vs MDCK-OCT2; ####*P* < .0001 vs MDCK-OCT2; §§*P* < .01 vs MDCK-MATE1; §§§*P* < .001 vs MDCK-MATE1; “no symbol” *P* > .05 (one-way ANOVA with subsequent Tukey-Kramer multiple comparison test).

Transport from the basal into the intracellular and the apical compartment is shown for all 5 putative biomarkers in Fig. 4. In comparison to MDCK-VC cells, the intracellular concentration in MDCK-OCT2 cells was significantly increased for serotonin, tyramine, 1-methylhistamine, and 5-amino valeric acid betaine, but reduced for 4-guanidinobutanoic acid. In MDCK-MATE1 cells, there was no significant difference in the intracellular accumulation compared to MDCK-VC cells for serotonin, tyramine, and 1-methylhistamine; however, there was a significant reduction for 5-amino valeric acid betaine and 4-guanidinobutanoic acid. Double-transfected MDCK-OCT2-MATE1 cells had significantly higher intracellular concentrations of serotonin and 1-methylhistamine than MDCK-VC cells, but significantly lower intracellular 4-guanidinobutanoic acid concentrations, whereas there was no significant difference between the cells for tyramine and 5-amino valeric acid betaine.

Transcellular basal to apical transport was not significantly increased in MDCK-OCT2 vs MDCK-VC cells for any of the 5 putative biomarkers. An increased transcellular transport was found for all 5 putative biomarkers in MDCK-MATE1 cells; however, it was not statistically significant for 5-amino valeric acid betaine and 4-guanidinobutanoic acid. Basal to apical transport of all investigated substances was significantly higher in monolayers of double-transfected MDCK-OCT2-MATE1 cells compared to MDCK-VC cells which supports in vivo data on OCT2/MATE1-mediated renal excretion and its inhibition by cimetidine in humans (Table 1).^16^

### 3.4 Specificity of intracellular uptake and vectorial transport

**Fig. 5:**
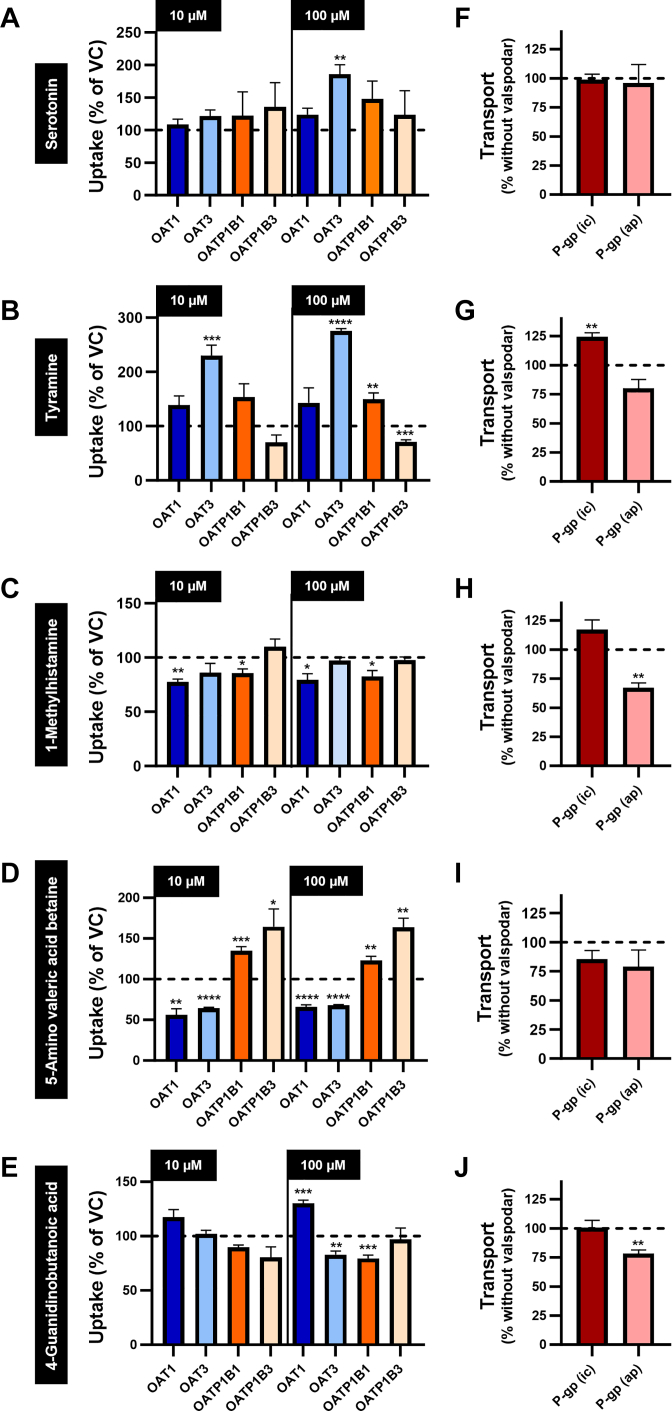
Uptake of 5 putative biomarkers for OCT2/MATE1-mediated DDIs in HEK cells overexpressing clinically important drug transporters and uptake and transcellular transport in Caco-2 cells. Uptake of (A) serotonin, (B) tyramine, (C) 1-methylhistamine, (D) 5-amino valeric acid betaine, and (E) 4-guanidinobutanoic acid (10 and 100 *μ*M) in HEK cells overexpressing OAT1, OAT3, OATP1B1, or OATP1B3 was measured after 5 minutes. Uptake into HEK-VC cells is set to 100% and is indicated by a dashed horizontal line. Intracellular uptake and transcellular transport from the basal to the apical compartment of (F) serotonin, (G) tyramine, (H) 1-methylhistamine, (I) 5-amino valeric acid betaine, and (J) 4-guanidinobutanoic acid (10 *μ*M) in Caco-2 cells was measured after 240 minutes in the presence and absence of the P-gp inhibitor valspodar (2 *μ*M). Uptake/transport without valspodar is set to 100% and is indicated by a dashed horizontal line. Data are shown as mean ± SEM resulting from 4 biological replicates. ∗*P* < .05 vs HEK-VC. ∗∗*P* < .01 vs HEK-VC or Caco-2 cells without valspodar. ∗∗∗*P* < .001 vs HEK-VC. ∗∗∗∗*P* < .0001 vs HEK-VC. “no symbol” *P* > .05 (two-tailed unpaired Student’s *t* test). ic, intracellular accumulation, ap, transcellular transport from the basal to the apical compartment.

Regarding the uptake transporters OAT1, OAT3, OATP1B1, and OATP1B3, only tyramine showed a significantly increased uptake ratio above 2-fold for OAT3 (Fig. 5). All other putative biomarkers had modest or not significantly different uptake rates between transporter-overexpressing cells and HEK-VC cells. In Caco-2 cells, the P-gp inhibitor valspodar significantly reduced basal to apical translocation of 1-methylhistamine and 4-guanidinobutanoic acid, but had no significant effect on serotonin, tyramine, and 5-amino valeric acid betaine.

### 3.5 Intracellular background in HEK, MDCK, and Caco-2 cells

Intracellular concentrations of the 5 endogenous compounds were determined at 0 minutes (ie, before the incubation with defined substance concentrations started) and after 5 minutes incubation in transport buffer without added substance. Serotonin, tyramine and 1-methylhistamine were not detectable at both time points, but significant amounts of 5-amino valeric acid betaine and 4-guanidinobutanoic acid could be measured in HEK cells.

**Fig. 6:**
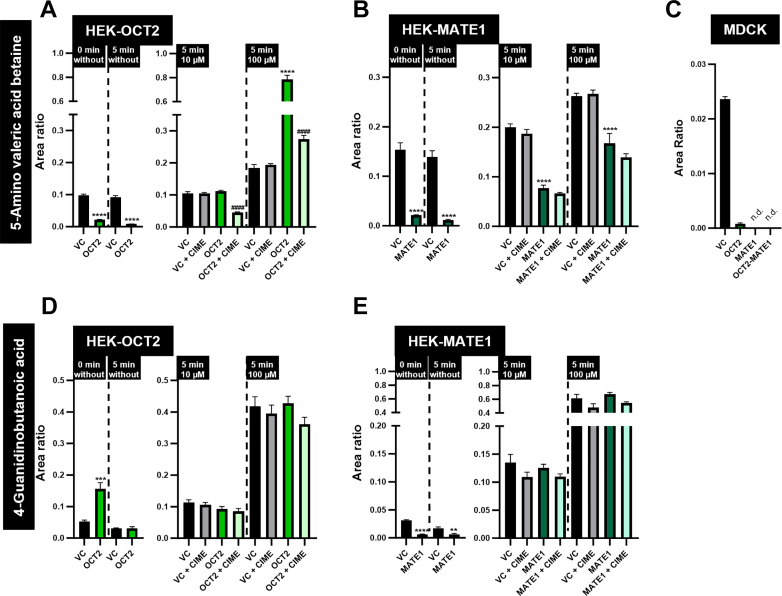
Intracellular background of 5-amino valeric acid betaine and 4-guanidinobutanoic acid in HEK and MDCK cells. Area ratios found intracellularly for 5-amino valeric acid betaine in HEK and MDCK cells (A, B, and C) and 4-guanidinobutanoic acid in HEK cells (D and E) found at 0 minutes and after a 5-minute incubation period in HEK cells and after 60 minutes of incubation without added 5-amino valeric acid betaine in MDCK cells. Data are shown as mean ± SEM resulting from 6 biological replicates in HEK cells and 16 biological replicates in MDCK cells. ∗∗*P* < .01 vs HEK-VC. ∗∗∗*P* < .001 vs HEK-VC. ∗∗∗∗*P* < .0001 vs HEK-VC. ####*P* < .0001 vs HEK-OCT2. “no symbol” *P* > .05 (one-way ANOVA with subsequent Tukey-Kramer multiple comparison test; only comparisons between HEK-VC vs HEK-OCT2 and HEK-OCT2 vs HEK-OCT2 + CIME are shown). 0 min without, intracellular concentrations found directly after cell culture; 5 min without, incubation for 5 minutes without added substance; n.d., not detected.

Interestingly, lower intracellular concentrations of 5-amino valeric acid betaine were found in HEK-OCT2 (Fig. 6A) and HEK-MATE1 (Fig. 6B) cells compared to HEK-VC cells at 0 minutes and 5 minutes without added substance, suggesting a role of both transporters for the handling of 5-amino valeric acid betaine. After incubation with 10 *μ*M of 5-amino valeric acid betaine, intracellular concentrations in HEK-OCT2 and HEK-VC cells were comparable. After incubation with 100 *μ*M, the intracellular concentration in HEK-OCT2 cells was significantly higher than in HEK-VC cells (Fig. 6A). Only a slight increase could be detected for both concentrations in HEK-MATE1 cells compared to HEK-MATE1 cells without added 5-amino valeric acid betaine (Fig. 6B). A significant effect of cimetidine was found only in HEK-OCT2 cells.

The intracellular 4-guanidinobutanoic acid concentration at 0 minutes was significantly higher in HEK-OCT2 cells compared to HEK-VC cells, but was reduced to the amount in HEK-VC cells after 5-minute incubation with transport buffer (Fig. 6D). In contrast, 4-guanidinobutanoic acid was significantly lower at both time points in HEK-MATE1 cells (Fig. 6E). Incubation with 10 or 100 *μ*M of 4-guanidinobutanoic acid did not result in any differences between HEK-OCT2 or HEK-MATE1 compared to HEK-VC cells.

In MDCK cells, an intracellular background of 5-amino valeric acid betaine could be detected in MDCK-VC and MDCK-OCT2 cells, whereas no 5-amino valeric acid betaine could be detected in MDCK-MATE1 and MDCK-OCT2-MATE1 cells (Fig. 6C). 5-Amino valeric acid betaine concentrations in MDCK-OCT2 cells were much lower than in MDCK-VC cells.

No intracellular background of any of the 5 putative biomarkers was detectable in Caco-2 cells.

### 3.6 Kinetic transport constants (K_m_ values) for OCT2- and MATE1-mediated transport of serotonin and 1-methylhistamine

**Fig. 7:**
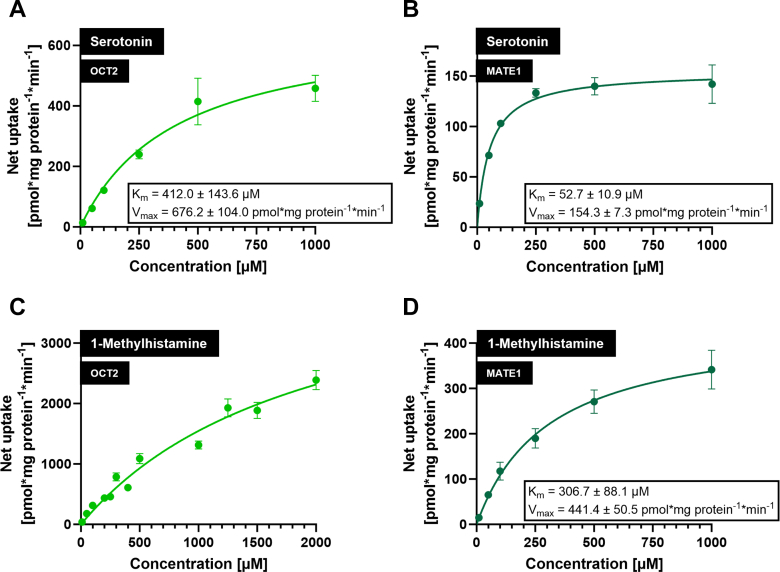
Concentration-dependent transport of serotonin and 1-methylhistamine in HEK-OCT2 and HEK-MATE1 cells. Determination of affinity constants (K_m_) and maximal transport velocities (V_max_) for OCT2- or MATE1-mediated uptake of (A and B) serotonin and (C and D) 1-methylhistamine at increasing substrate concentrations and an incubation time of 5 min. Data are given as uptake in pmol × mg protein^–1^ × min^–1^. Net uptake was calculated by subtracting values found in HEK-VC cells from values found in transporter-transfected cells. All data are presented as mean ± SEM resulting from 6 biological replicates.

The K_m_ values for OCT2- and MATE1-mediated serotonin and 1-methylhistamine transport were determined using concentration-dependent uptake experiments. Net uptake of serotonin and 1-methylhistamine was linear for at least 5 minutes for both HEK-OCT2 and HEK-MATE1 cells (data not shown). The K_m_ value of serotonin was 412.0 ± 143.6 *μ*M for OCT2 (Fig. 7A) and 52.7 ± 10.9 *μ*M for MATE1 (Fig. 7B). For 1-methylhistamine, concentrations up to 2000 *μ*M did not result in a saturation of net uptake in HEK-OCT2 cells (Fig. 7C); hence, calculation of the K_m_ value was not possible. A K_m_ value of 306.7 ± 88.1 *μ*M was determined for MATE1 (Fig. 7D).

## 4 Discussion

In this study, we used recombinant HEK and MDCK cell models, as well as parental Caco-2 cells and mass spectrometric quantification to investigate the transport of 5 endogenous biomarker candidates for renal DDIs mediated via the transport proteins OCT2, MATE1, and additional important drug transporters. The 5 candidates had previously been identified in a metabolomic analysis of blood and urine samples from a study with healthy volunteers. In that study, renal excretion of the 5 endogenous substances was significantly reduced after treatment with the OCT2/MATE1 inhibitor cimetidine.^16^ Our data support that OCT2/MATE1 mediate renal excretion of serotonin, tyramine, 1-methylhistamine, 5-amino valeric acid betaine, and 4-guanidinobutanoic acid and their inhibition by cimetidine appears as a major contributor to the observed in vivo effect. Transport mediated by the other investigated drug transporters OAT1, OAT3, OATP1B1, OATP1B3, and P-gp is much lower or completely absent compared to OCT2 or MATE1.

Serotonin is endogenously biosynthesized from the essential amino acid L-tryptophan, but minor amounts may also be absorbed from food.^25^ Urinary excretion was reported to be 0.21 ± 0.08 nmol/mL/mg creatinine in healthy subjects.^26^ In comparison to HEK-VC cells, we found high serotonin uptake rates in both HEK-OCT2 and HEK-MATE1 cells, whereas other transport proteins did not transport serotonin, except for OAT3 (100 *μ*M). However, the uptake ratio found at 100 *μ*M serotonin concentration for HEK-OAT3 cells (186%) is much lower than those found for HEK-OCT2 or HEK-MATE1 cells (6281%, Fig. 2A; and 1461%, Fig. 3A, respectively), suggesting only a minor role of OAT3 in renal excretion of serotonin. In polarized MDCK cells overexpressing MATE1 in the apical membrane, transport of serotonin from the basal into the apical compartment was considerably increased, which suggests a relevant role of MATE1 in the renal secretion of serotonin. Serotonin was described to be a substrate of OCT1 (K_m_ = 197 ± 42 *μ*M),^27^ which is another clinically relevant transport protein, primarily localized in the basolateral membrane of hepatocytes. Ma et al^17^ reported a K_m_ value of 539.1 *μ*M for OCT2, which is in the same range as our K_m_ value of 412.0 *μ*M (Fig. 7A). Of note, in our previous metabolomic analysis, administration of the OCT/MATE inhibitor cimetidine did not sensitively change serotonin plasma concentrations,^16^ which may suggest that other mechanisms eg, enzymatic degradation via monoamine oxidases or accumulation in platelets,^28^ could be of higher importance for systemic serotonin concentrations. However, data of our previous study indicate that not only renal excretion, but also the urine to plasma ratio of serotonin are sensitively decreased by cimetidine (log2-fold changes of –2.89 and –1.94, respectively).^16^ The K_m_ value of serotonin for MATE1 was determined as 52.7 *μ*M (Fig. 7B), which indicates a higher affinity of serotonin to MATE1 compared to OCT2. These findings, together with the high transport rates by OCT2 and MATE1 in the current in vitro study, support that renal clearance or renal excretion of serotonin may be sensitive markers for a risk of DDI via inhibition of renal OCT2/MATE1 and should be investigated in more detail in future studies.

Tyramine is a monoamine biosynthesized from L-tyrosine by fermentation or decay.^25^ The renal clearance after an oral administration of 400 mg tyramine was reported to be 260 mL/min in healthy volunteers, indicating relevant renal tubular secretion.^29^ Our results show that tyramine is a substrate of both OCT2 and MATE1. The uptake ratios found for HEK-OAT3 cells (230% when incubated with 10 *μ*M tyramine) are lower than what we found for HEK-OCT2 and HEK-MATE1 cells (3245% and 545%, respectively, when incubated with 10 *μ*M tyramine). However, considering a sensitive reduction of the excretion of tyramine into urine after treatment with probenecid in humans,^16^ OAT3 may contribute to its overall renal excretion in vivo. As dietary intake can constitute a major source for tyramine,^25^ it is necessary to pay attention to nutrition, if tyramine should be evaluated as a biomarker for DDIs in clinical studies. Taken together, the reduction of urinary excretion upon treatment with probenecid and the significant uptake in HEK-OAT3 cells suggest that tyramine may have limited specificity as a biomarker for OCT2/MATE1-mediated DDIs.

In humans, 1-methylhistamine is an endogenous metabolite of histamine.^25^^,^^30^ The mean urinary excretion is around 120 *μ*g/g creatinine, with a considerable variation between 30 to 250 *μ*g/g creatinine in healthy individuals.^31^ In our cell culture experiments, we found very high uptake/transport ratios in cells overexpressing OCT2 and MATE1 compared to the respective VC cells. In Caco-2 cells, there was a significant reduction of transcellular transport to 67% of the respective control if valspodar was added as a P-gp inhibitor, which points to 1-methylhistamine being a substrate also of P-gp. In the in vivo study using verapamil as a P-gp inhibitor, renal excretion of 1-methylhistamine was not affected.^16^ In that study, one single dose of 120 mg verapamil was administered to primarily inhibit intestinal P-gp. It is likely that this dose was too low to affect P-gp in the kidney.^32^ It would be interesting to investigate the effect of systemic P-gp inhibition on 1-methylhistamine renal elimination. In HEK cells, we found more than 10-fold higher uptake rates in HEK-OCT2 compared to HEK-MATE1 cells and in single-transfected MDCK cells, overexpression of OCT2 increased intracellular concentrations, whereas overexpression of MATE1 did not result in a significant intracellular reduction of 1-methylhistamine. Overexpression of both OCT2 and MATE1 still led to higher intracellular concentrations, which may point to a higher affinity of 1-methylhistamine to OCT2 than to MATE1. However, net uptake of 1-methylhistamine in HEK-OCT2 was not saturable (Fig. 7C). Therefore, we cannot directly compare the affinity to OCT2 with the affinity to MATE1 (K_m_ value of 306.7 *μ*M; Fig. 7D). Based on these data, 1-methylhistamine appears as a promising new biomarker candidate for renal DDIs mediated by OCT2 and/or MATE1. Future studies should investigate further properties of 1-methylhistamine, such as intraindividual and interindividual variation in plasma concentrations and renal clearance, circadian rhythm, susceptibility to other OCT2/MATE inhibitors, comparison between healthy and diseased populations, and others.

5-Amino valeric acid betaine is reported to be a microbial breakdown product of trimethyllysine, which can be found in vegetables and grains.^25^^,^^33^^,^^34^ To the best of our knowledge, renal clearance or urinary excretion of 5-amino valeric acid betaine in humans have not been investigated so far. Experiments in double-transfected MDCK cells show that there is coordinated OCT2/MATE1-mediated transcellular transport of 5-amino valeric acid betaine (Fig. 4D) in line with a likely role of these transporters for renal elimination of 5-amino valeric acid betaine. Intracellular background of 5-amino valeric acid betaine in HEK cells complicated interpretation of the uptake experiments. However, intracellular 5-amino valeric acid betaine in HEK-OCT2 cells incubated for 5 minutes with 10 *μ*M of 5-amino valeric acid betaine was increased at a much higher rate than in HEK-VC cells and the inhibition of this process by cimetidine suggests OCT2-mediated uptake of 5-amino valeric acid betaine (Fig. 6A). This is further supported by the significant increase of intracellular 5-amino valeric acid betaine at a substrate concentration of 100 *μ*M in HEK-OCT2 cells. A possible explanation for the low intracellular concentrations of 5-amino valeric acid betaine in HEK-OCT2 cells at the beginning of the experiments might be OCT2-mediated efflux of 5-amino valeric acid betaine when intracellular concentrations are higher than in the extracellular medium. This could be the case during the period of cell growth before uptake experiments were initiated. Previously, we reported a similar observation of OCT2-mediated efflux for trimethylamine N-oxide.^35^ We did not observe MATE1-mediated uptake in HEK-MATE1 compared to HEK-VC cells (Fig. 3D). However, we observed significantly lower intracellular concentrations of 5-amino valeric acid betaine in HEK-MATE1 cells in the uptake experiments both with and without addition of 5-amino valeric acid betaine (Fig. 3D and Fig. 6B). This may suggest highly efficient MATE1-mediated export from the cells. In vivo, we found a sensitive decrease of renal excretion (log2-fold change, –1.00) of 5-amino valeric acid betaine due to probenecid; however, we did not find relevant differences in the uptake ratios or transcellular transport compared to controls for the other transport proteins (in particular OAT1 and OAT3) investigated in this study (Table 1). Therefore, 5-amino valeric acid betaine is unlikely to be a substrate of OAT1/3 and the reduction of renal excretion is probably not due to probenecid inhibiting these transport proteins. Based on these results and the intense reduction of renal excretion we found in humans after administration of cimetidine (log2-fold change, –4.28 for renal excretion and –3.62 for renal elimination),^16^ 5-amino valeric acid betaine appears as a candidate biomarker for OCT2/MATE1-mediated DDIs. However, according to the data from monolayers of MDCK-OCT2-MATE1 cells, the extent of polarized, basal to apical translocation of 5-amino valeric acid betaine is considerably smaller compared to serotonin and 1-methylhistamine.

4-Guanidinobutanoic acid is a compound found in arginine metabolism, with a urinary excretion of 15 to 70 *μ*mol/g creatinine.^25^^,^^30^^,^^36^ In MDCK cells, we observed a highly significant basal to apical transport in MDCK-OCT2-MATE1 cells and a reduction of intracellular 4-guanidinobutanoic acid when MATE1 was overexpressed (Fig. 4E). This suggests that MATE1 acts as an exporter for 4-guanidinobutanoic acid and thus reduces intracellular accumulation and increases transcellular transport. Transcellular transport was most significantly increased when both OCT2 and MATE1 were overexpressed, which suggests that both transport proteins contribute to the transport of 4-guanidinobutanoic acid. This is in accordance with results from Nies et al^18^ suggesting a contribution of MATE1 to the transport of 4-guanidinobutanoic acid. Interestingly, the findings from the MDCK cells were not detectable in HEK-OCT2 and HEK-MATE1 cells. Similar to 5-amino valeric acid betaine, we also found relevant intracellular concentrations in HEK cells before uptake experiments started. However, there was no apparent transport protein-mediated uptake in comparison to HEK-VC cells for any of the transporters tested, even if intracellular concentrations at 0 minutes were taken into consideration (Fig. 6, D and E).

Some limitations of the present study need to be acknowledged. We focused our experiments on clinically relevant transport proteins that are known to be inhibited by the drugs cimetidine, verapamil, rifampin, and probenecid, as those were applied to healthy volunteers in our previously reported metabolomic analysis.^16^ These drugs may also have effects on other transport proteins or metabolic enzymes that were not investigated in the present study, because a comprehensive exploration of all putative metabolic enzymes for the biomarker candidates is out of scope of the current study. Upregulation of other transporters or metabolic enzymes due to the stable transfection of our cell models is unlikely, as they were used in numerous previous studies without any noticeable off-target effects. Nevertheless, as all of the investigated putative biomarkers are apparently substrates of OCT2 and/or MATE1, it is likely that the observed reduction of renal excretion in vivo after cimetidine treatment is due to inhibition of at least 1 of these 2 renally expressed transport proteins. In experiments using HEK-MATE1 cells, the transport direction of this proton-coupled export protein was reversed by adjusting extracellular pH to 8.0. Even though this is a well-established procedure, it may not be suitable for all kind of substrates, as intracellular and extracellular substrate recognition and affinities could be different.^37^ We addressed this by including polarized MDCK monolayers overexpressing MATE1 in our experiments. This led to the result of 4-guanidinobutanoic acid showing no apparent uptake in HEK-MATE1 (Fig. 3E), but increased transport across the apical membrane in MDCK cells overexpressing MATE1 (Fig. 4E). In case of 5-amino valeric acid betaine, we did observe a reduction in intracellular concentrations upon MATE1 overexpression, but no significantly increased uptake in HEK-MATE1 cells, which points to 5-amino valeric acid betaine to be preferably transported from the intracellular to the extracellular compartment by MATE1. For determination of concentrations of putative substrates, we used an LC-MS method, with which it is not possible to distinguish between molecules being intracellularly present at the beginning of uptake experiments (0 minutes) and molecules taken up during the 5-minute incubation period. For 5-amino valeric acid betaine and 4-guanidinobutanoic acid, we accounted for this limitation by taking intracellular concentrations at 0 minutes into consideration (Fig. 6). This may be overcome in the future by other experimental setups, eg, using radiolabeled 5-amino valeric acid betaine or 4-guanidinobutanoic acid.

## 5 Conclusion

In conclusion, the present study supports that OCT2/MATE1 mediates the renal excretion of serotonin, tyramine, 1-methylhistamine, 5-amino valeric acid betaine, and 4-guanidinobutanoic acid. Taking into account the reduction of the amount excreted into urine due to cimetidine in healthy volunteers, the results of the present in vitro experiments considering transport in HEK and MDCK cells and specificity with regard to other clinically relevant transport proteins (Table 1), serotonin and 1-methylhistamine are, in our opinion, the most promising candidates for further consideration as biomarkers for OCT2/MATE1-mediated renal DDIs.

## Conflict of interest

Martin F. Fromm has received consultancy fees from Boehringer Ingelheim and lecture fees from Janssen-Cilag. He has received third-party funds for research projects at his institution by Boehringer Ingelheim and Heidelberg Pharma Research GmbH. Martin F. Fromm and colleagues received an earmarked financial contribution for the first award of the MSD Germany Health Award 2021. Fabian Müller is an employee of Boehringer Ingelheim. All other authors declared no competing interests for this work.
